# Child and adolescent mental health services in a devolved healthcare system: a qualitative exploration of sustainable practices

**DOI:** 10.1186/s12961-023-00970-2

**Published:** 2023-04-05

**Authors:** Emily Banwell, Neil Humphrey, Pamela Qualter

**Affiliations:** Institute of Education, University of Manchester, Ellen Wilkinson Building, Manchester, M13 9PL United Kingdom

**Keywords:** Child and adolescent mental health, Mixed methods, Thematic framework analysis, Implementation sustainability, Implementation science

## Abstract

**Background:**

The transference of research evidence into routine healthcare practice remains poorly understood. This includes understanding the prerequisites of longer-term viability. The present study investigated the sustainable practices of GM i-THRIVE, a programme which reconceptualizes mental health services for children and young people (CYP) in Greater Manchester, United Kingdom. We aimed to establish whether a sustainable future was likely, and to identify areas of focus to improve that likelihood.

**Methods:**

The NHS Sustainability Model, typically completed as a questionnaire measure, was converted into interview questions. The responses of nine professionals, from a variety of roles across the CYP mental health workforce, were explored using inductive thematic framework analysis. Selected participants completed the original questionnaire.

**Results:**

Five themes (communication; support; barriers to implementation; past, present, and future: the implementation journey; and the nuances of GM i-THRIVE) and 21 subthemes formed the final thematic framework. Relationships with senior leaders and with colleagues across the workforce were seen as important. Leaders’ roles in providing meaning and fit were emphasized. Whilst training delivered the programme’s aims well, monitoring its dissemination was challenging. Widespread issues with dedicating sufficient time to implementation were raised. The flexibility of the programme, which can be applied in multiple ways, was discussed positively. This flexibility links to the idea of GM i-THRIVE as a mindset change, and the uniqueness of this style of intervention was discussed. To varying degrees, themes were supported by responses to the quantitative measure, although several limitations to the use of the questionnaire were discovered. Consequently, they were used to infer conclusions to a lesser degree than originally intended.

**Conclusions:**

Professionals involved with GM i-THRIVE reported many elements that indicate a positive future for the programme. However, they suggested that more attention should be given to embedding the core concepts of the model at the current stage of implementation. Limitations relating to its use within our study are discussed, but we conclude that the NHS Sustainability Model is a suitable way of guiding qualitative implementation research. It is especially valuable for localized interventions. The constraints of our small sample size on transferability are considered.

**Supplementary Information:**

The online version contains supplementary material available at 10.1186/s12961-023-00970-2.

## Background

The complete transference of research evidence into routine healthcare takes 17 years on average [1]. This lengthy duration contributes to a pessimistic larger picture, wherein only around half of evidence-based practices are ever implemented widely [2]. Despite this, intervention sustainability, a vital factor underneath the theoretical “implementation science” umbrella, has attracted relatively little research attention [3]. Defined as the “continued use of program components and activities for the continued achievement of desirable program and population outcomes” [4], sustainability is deemed “one of the most significant translational research problems of our time” [5]. Cohesive and definitive knowledge of the core factors underpinning longer-term viability is urgently required if interventions are to be sustainable from their outset, and ultimately, if the temporal research-to-practice gap is to be closed. The present study synthesizes some of the key issues raised in recent recognition of this dearth of sustainability research. To do this, a predominantly qualitative research strategy was taken, to explore the sustainable practices of GM i-THRIVE, a current children and young people’s (CYP) mental health intervention within Greater Manchester, United Kingdom.

**Fig. 1:**
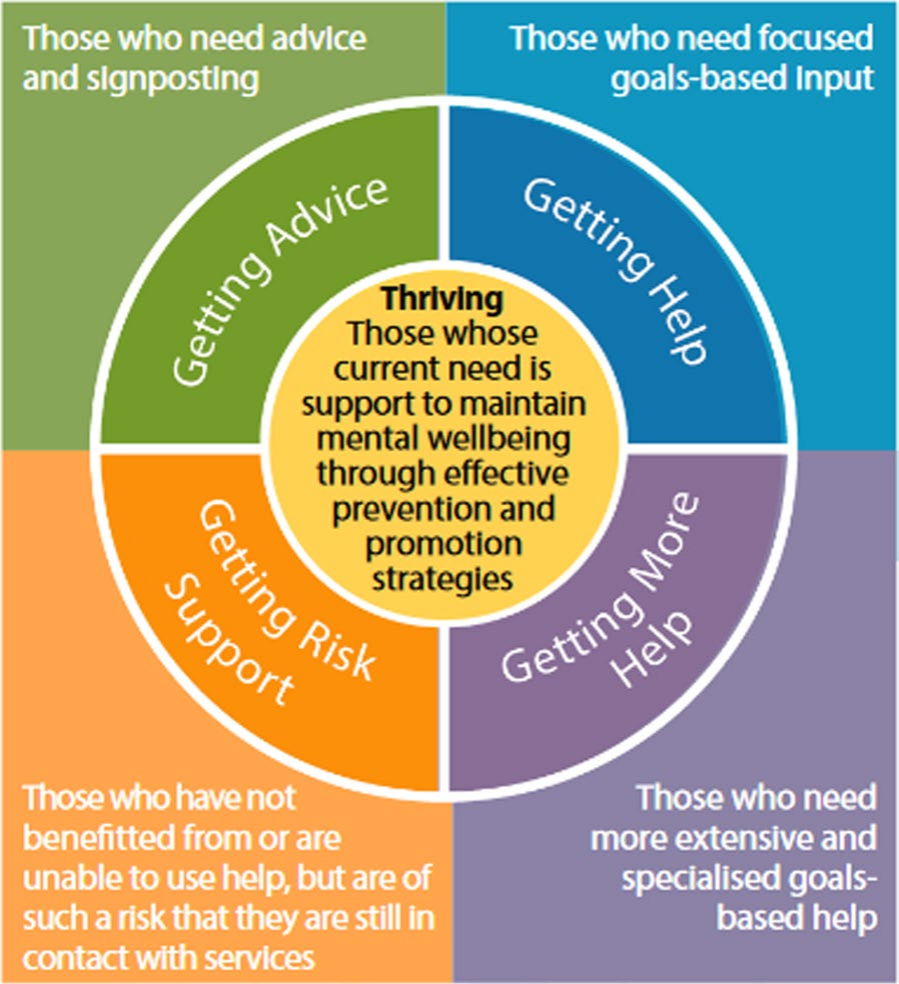
The five needs-based groupings of the THRIVE Framework for System Change [7]

Greater Manchester is one of the regions in England implementing the THRIVE Framework for System Change [6]. The nationwide initiative, known locally as GM i-THRIVE, aims to remedy the inadequacies of current CYP mental health services in the city-region. The THRIVE Framework migrates from the conceptually rigid and notoriously difficult-to-access tiered model of support traditionally used in the United Kingdom’s National Health Service (NHS) Child and Adolescent Mental Health Services (CAMHS). It aims to steer support services towards a service where shared decision-making and easy access are core. Figure 1 visualizes the THRIVE model’s needs-based groupings, under which support is decided upon collaboratively. It represents an inclusive, whole-system approach, where advice, support and care are allocated flexibly, as per *current* need, rather than by severity or mental health history. As a result, every CYP, including those considered “thriving”, can benefit.

THRIVE describes itself as a “shared language” and a mindset change, rather than a tangible “intervention”. Consequently, changing the way that mental health and service provision are conceptualized is key. Within Greater Manchester, an initial implementation period of 4 years (2018–2022) was allocated, followed by a short “embedding” phase, where implementation efforts continue. This overall duration clearly falls drastically short of the 17-year figure quoted as necessary for full implementation [1]. Although the evidence base is currently small, recent studies examining the effectiveness of “THRIVE-aligned” support found already-emerging positive changes [8–10]. However, focused investigation into the potential longevity of these changes is needed to assess their eventual impact. This is especially important given GM i-THRIVE’s short time frame. Post-implementation sustainability should be considered as early as possible in the implementation process for complex health interventions, so that protective strategies can be devised to overcome identified barriers [11]. This need, given that sustainable practice is such a crucial predictor of public health impact [12], was a fundamental driver of the present study.

### Rationale for the present study

We used an existing sustainability evaluation tool, the NHS Sustainability Model [13], to investigate GM i-THRIVE’s sustainable practices. It was designed by 250 subject experts and NHS staff, with 10 factors noted as key sustainability predictors [14]. These factors were then divided into three overarching areas: “process”, “staff” and “organization”. Brief explanations of those factors are provided in Table 1. Services using the model self-assess their own sustainability through a questionnaire measure (QM).

**Table 1:** Areas and factors that comprise the NHS sustainability model [13, 14]

Areas	Factors	Explanation
Process	Benefits beyond helping patients	Are there any other benefits to the change besides patient care, e.g. more efficient working practices, or reduced waste?
	Credibility of the benefits	Are the benefits obvious to, and describable by, everyone involved, and are they supported by evidence?
	Adaptability of the improved process	Can the new process withstand internal pressures? Can it meet ongoing needs without reliance on any individual/group/finance?
	Effectiveness of the system to monitor progress	Are there monitoring and feedback systems in place to be used beyond implementation, and is this information communicable?
Staff	Staff involvement and training to sustain the process	Do staff play a role in the design and implementation, and are they suitably trained?
	Staff behaviours toward sustaining the change	Are staff encouraged to express ideas, and do they think the new change is a better way of doing things?
	Senior leadership engagement and support	Are senior leaders trusted, involved, knowledgeable and responsible?
	Clinical leadership engagement and support	Are clinical leaders trusted, involved, knowledgeable and responsible?
Organization	Fit with the organization’s strategic aims and culture	Are the goals clear, and do they contribute to the overall aims of the organization? Have similar changes done well in the past?
	Infrastructure	Are staffing, facilities, policies and equipment suitable to sustain the implementation over time?

The NHS Sustainability Model is just one of several models and tools developed to research sustainability. Such tools have been developed to guide intervention setup, ensuring that sustainability is considered early on, and to determine whether an intervention is, or is likely to be, sustainable over the long term. The NHS Sustainability Model, whilst typically delivered through a questionnaire, has also guided qualitative research. Sustainability barriers and facilitators have been successfully identified by converting the items into an interview schedule [15]. Qualitative research methods are useful for exploring sustainability in smaller, localized interventions, where the statistical power needed for quantitative research is not present [3]. Despite capturing just a “snapshot” of an intervention at one time point, qualitative studies harness valuable stakeholder insights into the current practices, and impeding barriers, surrounding sustained usage [3]. The NHS Sustainability Model’s track record of methodological flexibility, combined with its NHS-centric design, was why it was ultimately chosen for this study. This choice also stemmed from lack of consistency across sustainability research. Whilst novel research approaches are often excellent ways of exploring ideas and appraising findings, this can make wider conclusions harder to draw, and collaborative progression of a field of research may be hindered. For unanimous progression, and more consistent measurement of implementation and sustainability progress and outcomes, greater reliance on existing models has been posited [5, 16]. Had we designed the present study based on our own perceptions of what were important elements of sustainability, not only would we would have contributed to the measure inconsistency noted as a limitation in the field, but important considerations might have been missed. Thus, the NHS Sustainability Model’s factors detailed in Table 1 were converted into questions in a semi-structured interview. To tailor the interview to GM i-THRIVE, three additional topics of relevance were added: (1) *Adaptability*: organizations should evaluate, respond and evolve to meet public health needs [17]. This is a vital consideration for GM i-THRIVE as a broadly applied intervention. (2) During implementation, staff should *reflect* upon their situation before the intervention was introduced [18]. Since THRIVE represents such a significant transformation from the tiered model, the facilitation of reflection is worthy of focused investigation. (3) A large portion of intervention dissemination in GM i-THRIVE is through *training* [19]*.* The emphasis on diffusing knowledge through organizational levels made this a vital interview topic. In addition to the interviews, selected participants completed the original QM, and their responses were corroborated, where appropriate, with the generated qualitative themes.

Adopting a purely post hoc evaluation approach that takes place only once official implementation phases have ended [11] can limit the scope of sustainability analyses, whereas combining retrospective investigation methods with those that offer the potential for ongoing improvement may increase their utility [20]. GM i-THRIVE’s implementation and embedding stages are summarized below. The plan was executed at a GM-wide level; however, it is clear from the outlined steps that the requirements and capacity of each locality and service within were considered. The data for the present study were collected from August to November of 2021, as Stage 4 (the reviewing of implementation projects and goals) was ending and Stage 5 (the continued embedding and monitoring of outcomes) was set to begin. This timing sits comfortably between a priori and post hoc research approaches, allowing investigation of sustainable factors occurring towards the end of, but still in the midst of, implementation [11].

Stage 1: Setup:

- Cross-sector approval of GM i-THRIVE established
- Stakeholder mapping undertaken
- Communication and engagement plan created.

Stage 2: Engagement, understanding and planning:

- Key goals and messages established, including locality contexts
- Staff, CYP and family consultations
- Service performance review: need, current practice, demand, clinical outcomes, current outcome measures, etc.
- Progress-monitoring method established
- Locality models of GM i-THRIVE created.

Stage 3: Capacity-building:

- Staff capacity, recruitment need and workforce development plan established
- Creation and training of locality leads
- THRIVE training for front-line staff and managers begun.

Stage 4: Implementation:

- Finalizing implementation outcomes in each locality
- Implementation projects designed, undertaken and monitored.

Stage 5: Embedding and sustaining:

- Learning collaboration established
- Embedding, sustaining and monitoring implementation projects.

Through this positioning, our objectives were to identify:

- Already-occurring sustainable practices
- Areas where sustainability could be enhanced during GM i-THRIVE’s “embedding” period.

## Methods

### Reporting guidelines

The production of this article was guided by the Standards for Reporting Qualitative Research (SRQR) [21]. A checklist of how these guidelines were met can be found in the *supplementary materials* (see Additional file 1).

### Researcher context

The authors were commissioned by the Greater Manchester Health and Social Care Partnership (GMHSCP) to evaluate GM i-THRIVE. As external researchers, our analyses were not influenced by vested interest. It is, however, prudent to acknowledge that impressions gained by the first author during regular meetings with GM i-THRIVE leaders and stakeholders may have inadvertently impacted the analyses and conclusions [22]. Despite this possible bias, the benefits of these afforded insights into working environments and practices undoubtedly outweighed the risk of biases attained through the same contact.

### Design

Our rationale for choosing a mixed-methods design was driven by complementarity [23]: meaningfulness and validity are improved by drawing on the strengths of qualitative *and* quantitative approaches. A pragmatic epistemological viewpoint was adopted, with the research and analysis methods selected purely for their practicality [24]. This paradigm emphasizes the value of useful and actionable research: higher-level abstraction is unnecessary, or even obstructive, when attempting to meet the aims of such studies [25].

### Setting

GM i-THRIVE is part of a wider devolution deal, drawn in 2016, between GMHSCP and the government of the United Kingdom. GMHSCP can now allocate resources to health and social care services, as per the needs of Greater Manchester’s 2.8 million city-region residents. Alignment of Greater Manchester’s CYP mental health services to THRIVE principles follows the philosophy of the wider devolution by aiming to provide an appropriate and diversified range of support and care.

The ethnically and socially diverse city-region of Greater Manchester (GM) is in North-West England, and comprises high-density urban areas, suburbs and rural locations. In total, 898 000 under-25-year-olds reside in GM, who are more likely to experience poverty and suffer poorer overall health outcomes than the average young person in the United Kingdom [26]. Ten locality boroughs (Bolton, Bury, Manchester, Oldham, Rochdale, Salford, Stockport, Tameside, Trafford and Wigan) make up GM, each with a GM i-THRIVE “lead” accountable for managing implementation across specialist NHS and other local services. These leads are overseen by a dedicated GM i-THRIVE team.

### Ethical considerations

Under NHS Health Research Authority guidelines, the present study was categorized as a “service evaluation”. The need for formal review by the NHS Research Ethics Committee, and the University of Manchester’s own research ethics committee, was therefore waived. However, informal approval was sought from the second and third authors (the first author’s supervisory team) and the study commissioner. Principles such as obtaining informed consent and ensuring secure data storage were followed.

### Participants

Nine participants were recruited with an opportunistic maximum variation strategy. Participants could respond to an email, sent by a gatekeeper within the GM i-THRIVE team, if they wished to take part. The key variation point of the strategy was that participants represented the levels of professional control and autonomy often present in healthcare organizations. Demonstrating this, an implementation sustainability study within nursing identified that staff had little authority over how they spent their time, how much freedom they had to innovate and, crucially, how much of an intervention they truly “received” [27]. Most factors that promote or hinder sustainability are influenced by the most senior staff, and exist outside the sphere of influence of those involved in practical implementation [27]. We consequently decided that a hierarchical range of “control” levels should be represented, encapsulating a full array of multi-organizational perspectives. Eventually, the sample comprised *n* = 2 participants with the highest amount of control: top-down implementers who make the key decisions that impact intervention sustainability. Those with moderate control (*n* = 4) were GM i-THRIVE locality leads. These are senior staff members with roles and responsibilities that are extraneous to GM i-THRIVE; however, they are responsible for implementation activity in their locality. Although they possess some degree of control over how they disseminate GM i-THRIVE, they sit centrally between receiving and providing guidance. The final group of participants (*n* = 3) are pure intervention recipients. Whilst they influence sustainable practices in a minor way, perhaps through suggestions and feedback, they are predominantly responsible for enacting their training and, as a result, have a low level of control. All of these participants worked with CYP within GM localities, as practitioners or therapists. Participants from six of GM’s locality boroughs were represented in the sample, and one participant (of low control) worked for a voluntary sector service that operates across seven boroughs.

### Data collection procedures

Construction of the semi-structured interview schedule was guided by items of the NHS Sustainability Model [13]. Additional questions added focus on adaptability, reflection and training. The schedule was tailored per interview, ensuring relevance to each participant’s professional role. A full copy of the interview schedule can be found in the supplementary materials (see Additional file 2). Owing to COVID-19 restrictions, interviews were held through online conferencing software. After verbatim transcription by the first author, each typed transcript was “member-checked”, which allowed participants to expand, amend or omit data that they no longer wished to be represented [28]. The QM was completed by all GM i-THRIVE locality leads (*n* = 4). These participants had the necessary level of expertise in the strategic inner workings and implementation-to-workplace translation of GM i-THRIVE for completion of the QM, for which the 10 factors (Table 1) were presented as a tick-box questionnaire, for participants to select their perception of sustainability progress.

### Data analysis

Transcripts were analysed using thematic framework analysis (TFA). TFA is a practical and transparent qualitative analysis method, deemed well suited to both health [29] and applied policy research [30]. Situated within the broader family of thematic analytical methods [29], TFA is suitable for research that, like the present study, has a priori issues that warrant exploration, a predetermined sample, and a limited research time frame [30]. Directive and actionable research outcomes are typically produced by TFA [31].

Verbatim transcripts were analysed by the first author, using the stages of TFA outlined by Gale et al. [29]. Firstly, familiarization and data immersion occurred by rereading transcripts, with key ideas coded line by line. Then, a working thematic framework was gradually developed to sort coded data. The interview schedule *can* be viewed as a pre-existing “framework”, as it undeniably guided the authors’ thematic thinking [30]. However, to ensure that the data were not forced into this framework, no deductive codes were used, allowing a predominantly inductive analytical approach. This approach differs from other qualitative studies guided by the NHS Sustainability Model [15] and, alongside the inclusion of three additional intervention-specific foci, represents the study’s aim of identifying the sustainability factors deemed the most important for GM i-THRIVE.

Following coding, data were “indexed”: sections of data corresponding to each theme were identified, then “charted” in a matrix using Microsoft Excel under headings from the newly developed thematic framework. Finally, the characteristics outlined in the charts were interpreted, and themes were refined and finalized. A portion of the final themes, codes, and extracts were “sense-checked” by the second and third authors, to enhance confirmability [32]. Ensuring that coding, charting and the underpinning thought processes make logical sense is a methodologically appropriate way of adding rigour to qualitative studies. Traditional “validity” is not a worthwhile goal of such research [33].

### Meta-inferences

Meta-inferences between the qualitative data and the QM were reached through an exploratory bidirectional approach. This means that, although the qualitative strand heavily predominates, and the quantitative results are presented separately, we aimed to conflate both sets of data iteratively when drawing study conclusions [34]. As the discussion section shows, however, our eventual ability to do this was hindered by several factors, which were presented as limitations of the QM’s use in this study.

## Results and discussion

The final thematic framework comprised five overarching themes and 21 subthemes, each pertaining to a factor influencing sustainability within GM i-THRIVE. For consideration alongside the analysis, Table 2 shows the thematic framework in its entirety, supported by example illustrative participant extracts. Please note that subtheme names are italicized within each theme analysis.

**Table 2:** Thematic framework, showing overarching themes, subthemes and example illustrative extracts

Overarching theme	Subtheme	Example illustrative extract (Participant number and professional control over the implementation process in bold)
Communication (9)	The dissemination of GM i-THRIVE (7)	“I'm responsible for coordinating our THRIVE partnership […] forum through which we engage, keep informed, work with collaboratively in terms of our strategy and planning work, sharing information. And we have very good attendance and engagement. I think that illustrates that people are bought into the agenda and know where to go.” ***(Participant 1, medium control)*** “That’s one of the biggest things, really, about that shared language. And I don't think we are there with that. I don't think that the wider workforce knows enough about it.” ***(Participant 6, low control)***
	The relationship between Greater Manchester and locality teams (6)	“I think that the GM team around THRIVE are really responsive. They celebrate the work that you do in a locality […] And they are a team that is quite approachable to problem-solve, so I think that's definitely a real help.” ***(Participant 2, medium control)***
	Accountability (7)	“I think some of them (locality leads) definitely have that personal accountability and responsibility, and you can see in some areas where it's really flourished […] And other areas, it's just starting to take off so there's definitely something about having the right people in place.” ***(Participant 4, high control)*** “A challenge will be moving forward, to support the practitioners and understanding what it means to them. Where do they fit within it? What do they already offer?” ***(Participant 9, low control)***
	Networking and joint working (7)	“I think that the whole THRIVE process has strengthened our services, links with CAMHS, we've got much stronger relationships. We're more connected and understanding of each other’s ways of working. And I think that sort of helps. I think just being clear on the different quadrants and what they mean for people.” ***(Participant 6, low control)***
Support (9)	The responsibility of locality leads (6)	“Being an advocate for the THRIVE principles, being that conduit in a system that tries to facilitate conversations between different organizations […] getting people to reflect on their own practices in accordance with the THRIVE principles, is one of the main responsibilities I'd say for a THRIVE lead.” ***(Participant 8, medium control)***
	The role of the Greater Manchester team (8)	“One of the key things is building relationships, that's it, and being open and helpful to people and trust, so bringing that familiar, having that relationship.” ***(Participant 4, high control)***
	Other senior support (4)	“What you couldn't pretend was that just having the fancy new diagram with THRIVE was going to solve that if you didn't sort out putting in the new and the extra services and the support to people. So I was somewhat cynical.” ***(Participant 5, high control)***
	Training and capacity-building (8)	“I don't think we had the right representation at the start […] It's the same people that always put their hand up, or always get nominated.” ***(Participant 3, medium control)*** “One of the things we probably struggle with is knowing how that training’s been progressed […] to say actually ‘have you used that training for your own practice?’ or ‘have you managed to train other people in your team?’ and understand kind of how far that’s gone.” ***(Participant 2, medium control)***
Barriers to implementation (9)	Workload (5)	“I feel like I've probably not got as much capacity to be able to truly focus on that all the time, which I think it could, it could be a role that someone could do full time, and still probably not be able to solve everything.” ***(Participant 2, medium control)***
	Conflicting priorities (6)	“The THRIVE leads are really passionate and keen, but maybe more limited with their capacity, because of their other work that they've got to do. And I think that's quite common across quite a lot of roles. But I think if you can then have that shared—ownership is probably the right word?—of continuing to implement it, bit by bit, I think that's more of a sustainable model as well.” ***(Participant 9, low control)*** “THRIVE has tried to change practices so that it's working smarter, not harder. In the short term, it might look as if there's a little bit more of an effort, and there's a bit of time that you need to take out to reflect on your service and build it in a THRIVE-like way.” ***(Participant 8, medium control)***
	The effect of “firefighting” on progress (4)	“When I look where we were at, say, 18 months ago, and what our aspirations were to do next, we’ve not been able to move on some of those things because it's about staff well-being, staff shortages, people being off sick, system changes, it's all been about firefighting and business continuity, sadly.” ***(Participant 1, medium control)***
Past, present and future: the implementation journey (9)	GM i-THRIVE was, and is, needed (7)	“What we were drawing on wasn't… It was things that were unsatisfactory really, actually a desire to move away from things that didn't work, and weren't as universally engaging or adaptable as what THRIVE actually is.” ***(Participant 1, medium control)*** “It’s a really good way to challenge decisions. It didn't mean that there was a different outcome. But it's always good to have the theory behind what we should be doing.” ***(Participant 7, low control)*** “We had what was previously the tiered model. Now, I don't think that framework is bad or wrong. And I think there's been a bit of a confusion with people saying ‘oh, THRIVE's so much better and THRIVE's much easier’. And it misses the point that the failure around the tiered model was about investment.” ***(Participant 5, high control)***
	A strong foundation (7)	“There's always been that commitment that THRIVE is the approach that we're going to take.” ***(Participant 2, medium control)*** “We've put that effort in, and now it's just about sustaining it, keeping it, keeping the momentum going.” ***(Participant 3, medium control)***
	Evidence of change (9)	“I think the biggest difference is just more conversations and less referrals.” ***(Participant 8, medium control)*** “I think, if it wasn't good, people wouldn't stretch it out anywhere, it wouldn't go as far as it's going now, if the effort was too much.” ***(Participant 4, high control)***
	Becoming routine (4)	“I think it has potential. But I think there needs to be a culture/thought shift amongst the whole system. And I think the challenge with that is how it aligns with other systems.” ***(Participant 6, low control)***
	Learning from reflection (7)	“Some observations I have made over time is that it's got to be more than a word. And I think that's key. It's got to be meaningful.” ***(Participant 9, low control)***
	Looking to the future (8)	“To make it sustainable, they need to sell this. It's that synergy of the bits coming together really, rather than just lots of training and people using pretty diagrams, which THRIVE does give us. But it's got to be more than that.” ***(Participant 5, high control)*** “It feels as if now we're at that pivotal, turning point where everyone's starting to get it.” ***(Participant 8, medium control)*** “Rethinking how we use the resources we've got now, for the best effect […] training and capacity-building is one of those, you can't just do that for a couple of years, and then hope that you’ve long-term sustained benefits, you’ve got to keep doing it.” ***(Participant 1, medium control)***
The nuances of GM i-THRIVE (8)	Unexpected consequences (4)	“A couple of people not understanding it, or thinking it was more than what it was.” ***(Participant 9, low control)*** “We never set out to look at that in the broadest context that we have. In a positive way, we certainly didn't set out with an ambition to apply the THRIVE concepts across much broader children’s services systems, and that's been a positive consequence.” ***(Participant 1, medium control)***
	Widespread change (8)	“Part of my portfolio encroached on the homelessness agenda. And I thought, you know what, we could use THRIVE here.” ***(Participant 3, medium control)*** “If I was to quote THRIVE every time I made a referral, I imagine that would help in terms of the outcome of that, and that might be something I can implement myself.” ***(Participant 7, low control)***
	Flexible application (5)	“It allows people to use it in different ways as well, but brings a commonality to it, so that shared language that everyone understands.” ***(Participant 4, high control)*** “I find that a positive and a negative, because you feel like you've got free run to do what's right for your area. But equally, you've not got anything to compare to whether you're on track.” ***(Participant 2, medium control)***
	How does the nature of THRIVE as a model influence implementation? (5)	“Because it's such a universal approach, and that it can apply to a lot of things, there's never like a definitive end where you say ‘we've officially embedded THRIVE’. It feels like it could always go on and on.” ***(Participant 2, medium control)*** “I think people thought THRIVE was a thing, rather than a set of principles and a framework. And that was the most difficult thing to overcome […] I think a framework takes longer. But I think there's many benefits to it, because it's more flexible for the system.” ***(Participant 3, medium control)***

Numbers in brackets represent the number of participants that contributed to each theme and subtheme

### Communication

The importance of open dialogue and widespread communication for *the dissemination of GM i-THRIVE* was raised by a range of participants. Well-attended core meetings, where ideas and strategies were shared, were reported. This demonstrates engagement with the programme, and active staff involvement in staying up to date. However, testimonies from participants with lower professional control questioned the reach of such platforms. Although they acknowledged that THRIVE featured in workplace conversations, participants wished for more formal briefings. One participant felt that those in the CYP workforce’s wider peripheries had not yet been sufficiently immersed in THRIVE’s conceptual framework. *The relationship between Greater Manchester and locality teams* was also raised as key. Several participants praised the helpfulness and approachability of GM i-THRIVE’s programme manager, who was easy to contact and keen to assist. Opportunities to share experiences and successes with this team were appreciated. In the literature, the role of managers as galvanizers who encourage innovation during implementation has been documented [35]. However, this may be just one factor of group efficacy, a factor more vital than senior management alone [36].

This efficacy within GM i-THRIVE may be fostered by *networking and joint working*. THRIVE’s common language enhanced communication between services, uniting the broad sector, and diverse peer networks within implementation helped inform improvement and navigate ambiguity in a contextually relevant way [37]. Participants also discussed the importance of knowing each other’s roles and practices, which is key for providing the cohesive service advocated by THRIVE. Accurate perceptions of others’, and one’s own role in implementation is crucial when aligning an intervention with already-existing organizational practices. Misunderstandings may be detrimental to sustainability [38].

Participants thought that if staff felt *accountable* for their role in implementation, the benefits would be more effective. Communicating and evidencing personal meaning to implementing staff, by demonstrating where their own roles fit and emphasizing the differences that they could make, is vital. It was also important for busy GM i-THRIVE locality leads to feel accountable. Leads who could seamlessly integrate THRIVE work into their other responsibilities reportedly made the quickest progress. A review of sustainability tools found that accountability for intervention delivery featured in slightly over half of models [39]. This suggests that whilst its importance may differ per intervention, in the case of THRIVE, varied application across job roles means that knowing how it applies to one’s own work, and feeling responsibility for this application, is vital.

### Support

Staff at all levels reported the value of providing and/or receiving support across the implementation process. *Locality leads saw their key responsibility* as galvanizing and steering, rather than direct implementation. Although a lack of clinical knowledge was reported, they perceived themselves as conduits between the Greater Manchester team and locality staff. Leads deliver key messages, and encourage reflection and conversation. *The role of the Greater Manchester team,* as reported by a member of this team, is, again, to enable. They saw themselves as facilitating relationships and promoting familiarity around THRIVE. Other participants described this team as a friendly central point of contact. Their support and direction were vital, and as such, should not be withdrawn in the near future. The key point raised under the subtheme “*other senior support”* was the necessity of senior buy-in. It was essential for service leaders, and those in commissioning roles, to be convinced that THRIVE could make positive changes. One such participant admitted initial cynicism, stating that other issues, such as staffing and service provision, needed to be resolved before THRIVE could be properly received. Cynicism is an identified barrier to organizational change and reform across all professional levels [40–42], with manager cynicism influencing employee commitment to change [40]. The senior leadership QM factor, although deemed one of the most important [13], produced mixed results. However, owing to the ambiguity of QM guidance, exactly who leads were referring to when answering is unclear in this instance.

*Training and capacity-building* was raised many times. Whilst participants reported that, generally, the training delivered THRIVE’s aims well, an appropriate foundation of suitable trainees, who possessed the correct level of background knowledge, was not in place at first. This meant that the same individuals attended all sessions, inevitably limiting the training’s reach. Keeping track of whether and how training is embedded and disseminated was also reported as challenging, with no formal mechanism in place to assess this. Having enough trained staff is an important predictor of intervention outcome [43], yet the factors underpinning the transfer of new skills to behaviour remain poorly understood [44]. Our findings further demonstrate the need to monitor this, especially considering that within GM i-THRIVE, a diverse group of staff receive identical training. Understanding and application inevitably vary greatly, requiring tailored monitoring. Training and progress-monitoring featured in 76% and 84% of sustainability frameworks, respectively [39], demonstrating their near-universal reputation as important for long-term viability. This observation is particularly noteworthy given that studies directly investigating THRIVE-aligned support have suggested that better outcome [8] and performance monitoring [9] are needed for full impact. In the QM, the “staff involvement and training” item was generally scored positively, yet “effectiveness of the system to monitor progress” revealed mixed opinions. These echoed sentiments from the interviews: whilst training content was good, dissemination had room for improvement.

### Barriers to implementation

Several barriers were discussed. A high *workload* limited the hours that locality leads could dedicate to GM i-THRIVE. Leads mentioned that although the ethos of the programme allows localities to work within the constraints of their resources, a full-time role would still be needed to dedicate a required amount of attention. One participant suggested creating this role to make GM i-THRIVE more sustainable. It was clear that staff at all levels handle *conflicting priorities*. Whilst GM i-THRIVE has created enthusiasm, implementation must fit alongside other tasks and roles. Greater sharing of responsibility was suggested, plus promoting the idea that whilst initial investment of time and effort is needed, this will eventually result in more streamlined work practices. The related *effect of “firefighting” on progress,* particularly in the context of COVID-19, was frequently mentioned. Dealing with urgent challenges as they arise, to maintain equilibrium, often take the fore. As a result, the time and energy needed to innovate and champion new strategies becomes limited. In 2020, the Academy of Medical Sciences produced a report outlining the lack of capacity allocated to research within the NHS [45]. This limits scientific innovation, and the subsequent implementation of evidence-based care. From this report, it is hoped that links between health and academia will be strengthened, and that NHS staff can dedicate more time to developing and incorporating evidence. The “infrastructure” factor of the QM connects with this theme, but covered facilities, policy and equipment in addition to staffing. This may be why reports were mixed.

### Past, present and future: The implementation journey

This theme includes discussions covering a range of implementation time points, which are grouped together under one theme because of the chronological story they tell. A variety of opinions on the extent to which *GM i-THRIVE was, and is, needed* were expressed. The strict tiered model previously used meant that THRIVE’s diversified and flexible support options were appreciated. Although better outcomes were not always evident, having the framework available when making decisions was valuable. Despite these observations, THRIVE should not be seen as a “quick fix”. One participant believed that the shortcomings of the tiered model are due to investment allocation rather than the nature of the model itself.

Locality leads felt that their own early involvement in implementation had built a *strong foundation* for the process. They felt that their locality had had a head start in making and sustaining change. These leads reported commitment to the framework and had no doubts about its suitability. This commitment has built enthusiasm and motivation, and now that this solid foundation is in place, sustainability is a priority. Desire for improvement has been identified as a key driver of change [3, 46], and staff commitment is important for overall implementation [47], fidelity [48] and maintaining adaptations [49]. However, sustaining commitment can be challenging when conflicting priorities are present [50].

Whilst the previous two subthemes scoped the situation before GM i-THRIVE, and the starting points that the localities were working with, the next subthemes discuss what progress looked like to participants at time of interview. Various examples of *evidence of change* were mentioned. Participants reported that there had been a gradual shift to THRIVE’s shared language, and they felt that the process of allocating mental health provision had been simplified. Referrals are now fewer, and those made are handled more appropriately. Many participants focused on aspects of networking, stating that services are more connected and that they feel more supported. Natural dissemination was also mentioned, including the idea that if GM i-THRIVE were not widely perceived as beneficial, it would not have developed to its current extent. Being able to demonstrate tangible advantages is widely reported as necessary for sustainability [39]. This means that GM i-THRIVE staff, upon recognizing that changes have already taken place, may feel more motivated to continue, or even bolster, their efforts. “Credibility of the benefits” in the QM was rated unanimously, in that although some benefits were clear to see, there was room for improvement. Evidencing and documenting THRIVE’s improvements should therefore continue into the next phase of implementation.

Whilst familiarization with THRIVE was reported as initially slow, it is steadily *becoming routine.* The model now features in daily working lives, though some mentioned that a wider culture and mindset shift is still forming, and that embedding this change is key for the future. Continuing to assess fit with current practice across the sector is vital, and this assessment should consider the distinct yet intricately related conceptualizations of technical, cultural and political fit [51]. Participants also felt that to move forward, THRIVE must shift from an abstract to a tangible concept for every involved member of the workforce. They must be given the tools to think about what THRIVE means to their role, and where their role fits into the wider implementation. This is an example of how *learning from reflection* can build sustainability [18], improve future outcomes and make an intervention meaningful in all contexts [52]. “Fit with the organization’s strategic aims and culture” was the best-scored item of the QM, suggesting that THRIVE’s strategic aims align well with those of localities.

Reflecting in this way also facilitates the proposal of “next steps” that can be applied when *looking to the future.* One locality lead felt that after 3 years of implementation, a significant turning point had just been reached. Understanding and familiarity had become sufficiently deep and widespread, providing a suitable basis for bigger changes. This was echoed by acknowledgement that true sustainability will involve the broad synergy of professionals and their knowledge. Efforts invested in strategies such as training must therefore continue if benefits are perceivable in the long term.

### The nuances of GM i-THRIVE

A key *unexpected consequence* of GM i-THRIVE related to misunderstanding its nature. On occasion, prior expectations of what the framework would offer were not met. Some anticipated greater changes that would rapidly resolve issues, whilst others expected a defined “intervention” instead of a mindset change. THRIVE’s shared language has also been interpreted differently by different individuals. However, an unexpected yet positive outcome was the broadness of THRIVE’s relevance. The diverse systems against which it can be applied far exceeded initial plans. Interventions with surplus value are generally viewed positively [53, 54]. “Benefits beyond helping patients” was mostly well-rated in the QM, which supports the interview data.

This links closely to the subtheme of *widespread change.* THRIVE as a mindset shift has allowed staff to apply their new knowledge to other responsibilities outside CYP mental health. This *flexible application* also allows it to be used in numerous ways, rather than by following a regimented set of instructions. Despite this flexibility, the framework still brings commonality to the sector, with principles that are understandable by all. However, a negative side to flexibility can result from localities applying the principles non-uniformly, which can present a challenge to comparison, “best practice” and progress-monitoring. Implementing so that the intervention remains recognizable, whilst simultaneously ensuring that guidelines are flexible and broadly applicable, is widely reported in the sustainability literature as a challenging balance to strike. Many researchers exploring implementation fidelity and adaptability have eventually concluded that these two concepts are a false dichotomy [55, 56]. Not only are adaptations intrinsically necessary for many interventions, but actively encouraging them can make full adoption more likely [56]. Many sustainability researchers have used ecological theory to explain that implementation inevitably involves constant adaptation according to constantly changing internal and external contexts [11, 57]. Whilst the importance of implementation fidelity varies per intervention [16], THRIVE clearly requires adaptation to meet the diverse needs of CYP and locality staff. Pragmatically establishing what “best practice” looks like, identifying the core components of THRIVE, and considering what each of these looks like per locality context is suggested [3, 16]. Once determined, other variations should not be seen as problematic deviations from the model’s core ethos [55].

Several nuances relating to *THRIVE as a model influence implementation.* Several participants mentioned the positioning of THRIVE as a framework, and as a set of principles to work with. THRIVE has been described within the literature as a “paradigm shift” [58], a “re-design” [10] and a “conceptualization” [7]. Whilst some consider implementation complete if its core elements are sustained over time [16], participants felt that THRIVE’s nature does not demarcate a clear endpoint to implementation. Reinforcement, training and embedding will be necessary for years to come. Although explaining THRIVE in these terms has been difficult, one locality lead said that whilst frameworks do take longer to implement than other types of intervention, the advantages are likely to be greater. Ensuring that mindset changes are as widespread as possible before the implementation period expires is therefore essential, given that these changes are unlikely to be complete by this time. The factor referring to adaptability within the QM discusses whether implementation can withstand removal of support. The findings suggested that GM i-THRIVE had not yet reached a point in its sustainability journey where such support could be fully withdrawn.

### *Strengths andlimitations:*

A key strength is that an existing sustainability framework guided this study’s design. This contributes, albeit in a small way, to alleviating the use of inconsistent measures within the field [5, 16]. The NHS Sustainability Model added evidence-based structure to our investigation of sustainability within GM i-THRIVE [15]. The model’s factors, of known relevance to sustaining NHS-centred interventions, were an important starting point for our interviews, and our predominantly qualitative approach enabled substantially deeper discussion of sustainability than the QM alone.

Despite using the model to develop the interview schedule, analysis was inductive. In a similar study, the model’s factors formed a deductive coding structure [15]. Yet, as extra topics were covered to improve application to GM i-THRIVE, forcing our transcripts into a strict framework was inappropriate. Rather, the most salient topics developed the thematic framework. This inductive approach does, however, warrant discussion as a study limitation. The thematic framework’s lack of direct match with the NHS model inevitably led to difficulties corroborating it with QM completions. The lead author therefore used their judgement when linking responses. As a result, some QM items were not cross-referenced if they did not fit. Whilst this questions the existence of true meta-inferences, it is worth reiterating that the QM formed only a small part of this research. They were merely used to elaborate upon the qualitative findings [34]. Owing to the poor ability to cross-reference, the eventual role that the QM played was even lower than originally intended.

The fact that themes did not perfectly match the model’s factors may appear surprising given that it guided the interview. However, this essentially portrays the NHS Sustainability Model, particularly when converted to interview, as comprehensive enough for participants to discuss what *they* deem important for sustainability, within their implementation. In fact, a model that includes the “right” sustainability constructs for *every* intervention does not, and certainly should not, exist, owing to the unique nature and contexts of each [39]. An inductive exploration is consequently important. Additionally, the advantageous peri-implementative standpoint of this study allowed us to look backward as well as forward. The positioning was especially opportune, as in 2021, most of the initial work of planning, locality-assessing and workforce-building had already begun. This paved the way for the embedding of core concepts and ideas to build understanding, which was incidentally perceived by participants as a vital “next step”. Considering this, it is crucial that the GM i-THRIVE team continue to monitor these sustainable practices and outcomes. After all, sustainability should not be considered a single outcome that only needs to be measured once [39]. Instead, it is a dynamic process that is highly sensitive to changes in the contexts in which it occurs [3, 11].

Although rich insights were obtained, we acknowledge the impact of the small sample size on the representativeness and transferability of our findings. Despite attempting a maximum variation strategy, the final sample was taken opportunistically. From points raised by participants, we attribute our recruitment difficulties to time constraints across the sector. Despite this, the locality boroughs participants worked in were well represented across the sample, which brought a diverse range of experiences with the implementation to the interviews. All participants contributed to each theme, with all subthemes containing views from at least four participants (see Table 2). An even smaller number of perspectives were included in the QM. However, the locality leads were the only participants with an appropriate level of strategic knowledge for meaningful completion. Even then, difficulties and ambiguity when answering some factors were informally raised. One may question why, given the small pool of participants, and the issues raised with answering and using the data, we chose to include our QM findings within this paper. Whilst completely omitting use of the measure from this report would have been a valid decision that would have perhaps presented a more streamlined set of findings, we decided that the limitations to the QM’s use within this context should be shared. Such presentation may indeed help other researchers designing similar studies. This transparency is important given that measure replicability and consistency is, as reported in the introduction, lacking in the implementation sustainability research field. These limitations also highlight the value of qualitative approaches for translating the measure into a more accessible, flexible and meaningful format. Through these methods, participants with any level of strategic knowledge can express insights into implementation sustainability. Questions can be worded appropriately for each participant’s role, whilst the essence of each of the model’s questions is still captured. Finally, the nature of THRIVE as a framework, and that it is not a tangible, directly applicable intervention, limits transferability to comparable innovations. This is especially true under the unprecedented context of the COVID-19 pandemic. Further qualitative investigations using the NHS Sustainability Model, with other types of intervention, are hence recommended.

## Conclusions and implications

This study took a predominantly qualitative approach to exploring sustainable practices within GM i-THRIVE. Using an interview schedule that was developed by combining the NHS Sustainability Model with intervention-specific additional points of interest, the qualitative data were summarized with five overarching themes. We tentatively conclude, overall, that GM i-THRIVE, as the implementation moves into its “embedding” phase, should look forward to a sustainable future, providing that attention is given to a number of key points that were gleaned from the thematic framework. Firstly, we found that senior staff played a vital part in facilitating GM i-THRIVE, and that locality leads should continue to ensure that staff understand exactly what the principles mean to them. Monitoring knowledge dissemination is therefore a crucial consideration, and enhancing this knowledge and familiarity will be key for embedding long-lasting change in all localities. Although this is a problem rooted in wider systemic issues, a culture of “firefighting” has limited implementation. Sustaining commitment to the key messages and practices of GM i-THRIVE is vital under these circumstances, so that they do not get lost or forgotten in favour of older methods that are familiar and easy, yet unhelpful in the long run. Clear strategies for how to overcome this may need to be devised. Finally, whilst the adaptability of THRIVE principles enhances its reputation, the length of time that it takes to fully implement and sustain a mindset change like THRIVE should not be underestimated. Even towards the end of the 4-year initial implementation period, the nature of THRIVE, as a framework of principles rather than a stand-alone concept, means that the process is likely to take a good deal longer. Accordingly, measures to enhance sustainability, as indicated by this study, are going to be key. From a methodological standpoint, the study provides a helpful example of how the NHS Sustainability Model can be used to stimulate qualitative discussion through interview, which is particularly valuable for smaller-scale interventions such as this. Although THRIVE is a nationwide initiative, the local application in Greater Manchester, over a limited number of sites, makes this relevant here. Further research is needed to validate the model’s applicability to other types of intervention when using it alongside qualitative methods.

## Supplementary Information

**Additional file 1.** SRQR guidelines checklist.

**Additional file 2.** Interview schedule.

## Data Availability

The semi-structured interview schedule can be found within the supplementary materials. The dataset generated during the current study is not publicly available owing to privacy concerns due to potentially identifiable information within the interview extracts. This is in accordance with the research governance policy of the University of Manchester. However, the data may be available from the corresponding author (EB) on reasonable request.

## Abbreviations

CAMHS: Child and Adolescent Mental Health Services
CYP: Children and young people
NHS: National Health Service
QM: Questionnaire measure
