# Supplementary material for: Child and adolescent mental health services in a devolved healthcare system: a qualitative exploration of sustainable practices
Source: Health Res Policy Syst. 2023 Apr 5;21:27. doi: 10.1186/s12961-023-00970-2 (PMC10075492; doi:10.1186/s12961-023-00970-2)
Supplement: Supplementary file 2 — Additional file 2. Interview schedule. [file 12961_2023_970_MOESM2_ESM.docx]

**A qualitative exploration of sustainable practices within GM i-THRIVE – Interview schedule:**

Senior staff:

Hello, thank you for agreeing to do this interview. As you’ve probably gathered, the main focus of the interview is to talk about how well THRIVE is setting itself up to be sustainable in the long run, and the processes that are in place to do this. Your perceptions of how things are going will be really useful to hear, so your honest opinions about how things are going, and your suggestions for what you feel can be improved will be really appreciated.

I’m sure you won’t, but try not to divulge any confidential information about anyone that you mention, if you do mention another person in whatever context. If you do mention any names, they will be anonymised in the transcript. And obviously for the evaluation I want to hear the good and the bad, so feel free to be as honest as you can be about your experiences!

I just want to reinstate that you are free to stop and/or withdraw from the study at any time, if you just let me know, we can stop. If you do decide to continue, this Zoom call will be recorded. Zoom also automatically transcribes our conversation, and once I’ve used your recording to check this generated transcript for accuracy, this recording, both audio and visual, will be deleted. But, if you do want to turn the camera off now so that your face isn’t recorded at all, you can do that if you wish? It’s up to you. Do you have any questions?

Are you happy to begin? I will start the recording if so.

Others:

Hello, thank you for agreeing to do this interview. The main focus of this interview is to talk about THRIVE – what you know about it, what you think about it, and how it’s influenced your daily procedures at work – that kind of thing, really. Your honest perceptions of how things are going on the ground will be really useful to hear, and your suggestions for what you feel can be improved going forward will be really appreciated.

Try not to divulge any confidential information about anyone that you mention, if you do mention another person in whatever context. If you do mention any names, they will be anonymised in the transcript. I just want to make it clear that even though I am working on an evaluation of i-THRIVE which is funded by them, my position is completely impartial, so please feel free to be as honest as you can with your experiences: we want to hear the good and the bad, and there won’t be any negative consequences of you doing this!

I just want to reinstate that you are free to stop and/or withdraw from the study at any time, if you just let me know, we can stop. If you do decide to continue, this Zoom call will be recorded. Zoom also automatically transcribes our conversation, and once I’ve used your recording to check this generated transcript for accuracy, this recording, both audio and visual, will be deleted. But, if you do want to turn the camera off now so that your face isn’t recorded at all, you can do that if you wish? It’s up to you. Do you have any questions?

Are you happy to begin? I will start the recording if so.

**Staff:**

*Top-down implementors:*

- So, could you start by outlining your job title and role?
- What is being done to ensure that locality staff, at all levels, feel involved in the roll-out of i-THRIVE?
- Which types of professionals, in terms of both job roles and seniority levels, have been involved in the design and planning of new ways of working?
- **Prompts for the above two questions:** what has their involvement looked like? To what extent have the different levels across services been involved? Do you feel that this level of involvement has been sufficient across all levels? Why/why not?
- **Only ask if not covered above:** How are staff encouraged to express their ideas or concerns throughout the change process, and how is their input being taken on board?
- **Prompt**: are there, for example, any regular meetings where staff can express their thoughts, and have actioned/un-actioned changes fed back to them? Are there also any, perhaps more informal mechanisms by which feedback can be given?
- How were the THRIVE leads chosen? In what ways are they “visible”, approachable, and well known to those on the ground?
- **Prompt:** Do staff know who the lead is? How to contact them?
- To what extent do you feel that the THRIVE leads feel a sense of personal responsibility for the change process in their locality?
- **Prompt:** Can you give an example of how a lead has shown that they do/do not? (Do/do not depends on response to the above question)

*THRIVE leads:*

- What is your main job role/other work-related responsibilities, aside from being the THRIVE lead for your locality?
- In your own words, what does being a THRIVE lead entail?
- How are staff in your locality encouraged to express their ideas or concerns throughout the change process, and how is their input being taken on board?
- **Prompt**: are there, for example, any regular meetings where staff can express their thoughts, and have actioned/un-actioned changes fed back to them? Are there also any, perhaps more informal mechanisms by which feedback can be given?
- What steps do you take to ensure that you are “visible”, approachable, and well-known to the staff implementing THRIVE in your locality?
- **Prompt:** Do staff know you and your role? Are there mechanisms by which they can contact you?
- How satisfied do you feel with the support that you have been given as a THRIVE lead? Is there anything in particular that has really helped? Or, what do you feel could help you more?
- **If not made clear above:** Do you feel you have actively had to seek support yourself, or are there formal processes or meetings that actively offer you it?

*Other staff members:*

- What is your job role?
- Tell me about how you have been involved with the implementation of i-THRIVE?
- **Prompt:** How has it changed your daily working practices?
- Are you able to express your ideas and concerns about the i-THRIVE implementation process? How? Do you feel that these are taken on board?
- **Prompt**: are there, for example, any regular meetings where you can express your thoughts, and have changes fed back to you? Are there also any, perhaps more informal mechanisms by which you feel you can give feedback?
- On a related note, has a system been made clear to you through which you can approach someone with any concerns or queries that you have about the implementation?
- **Prompt:** Do you know who to contact?
- In your opinion, how has i-THRIVE changed the way you do things, compared to before it came about?
- **Prompt:** Personally, do you feel that it is worth preserving? **(If not)** what changes do you think could be made to make it a worthwhile intervention to keep?

**Process:**

*Top-down implementors:*

- In your opinion, what are the most important elements of THRIVE that you feel should be kept and sustained?
- **Additional:** What barriers do you foresee to being able to sustain them?
- In addition to the outcomes of THRIVE that directly relate to supporting CYP, what other changes, particularly in workplaces and within teams, have you seen as a result of implementing THRIVE? **Prompt:** for example has it made people’s jobs easier or harder? Have processes been streamlined? Has duplication been avoided? **Ask to give examples if says yes or no.**
- From your perspective, what are the key differences that you think staff notice, or will notice, in their working lives as a whole?
- Do you believe that THRIVE is overall well-placed to meet the ongoing needs of CYP? How are the changing needs of GM currently being monitored and how is this monitoring acted upon?
- More importantly, since we’re talking about sustainability, what mechanisms will be in place to monitor changing needs of CYP, localities, and staff, *beyond* the formal implementation phase? This can be both formal and informal mechanisms.
- More generally, what long terms plans are in place for sustaining and/or widening the programme?
- To what extent has i-THRIVE been impacted by COVID-19? Do you think, realistically, that it has impacted how well the principles of THRIVE have been delivered? How so?
- **Prompt:** Tell me about what changes have been made to the training academy.

*THRIVE leads:*

- In addition to the outcomes that directly relate to supporting CYP, what other changes, particularly in workplaces, and within your teams, have you seen as a result of implementing THRIVE? **Prompt:** for example has it made people’s jobs easier or harder? Have processes been streamlined? Has duplication been avoided? **Ask to give examples if says yes or no.**
- From your perspective, what differences do you think staff notice, or will notice, in their working lives as a whole?
- Are you optimistic that THRIVE will continue to meet the ongoing needs of CYP, in particular, beyond the formal implementation phase?
- More generally, do you know of any long terms plans that are in place for sustaining and/or widening the programme?
- To what extent has i-THRIVE been impacted by COVID-19 in your locality? Specifically, do you think, realistically, that it has impacted how well the principles of THRIVE have been delivered? How so?

*Other staff members:*

- What differences in your working life have you noticed, that you could attribute to the introduction of THRIVE? **Further question**: if none, do you think you will in the future?
- Has patient care/interactions with CYP (change depending on person’s specific role) changed much as a result of THRIVE? What do you think has changed the most? What has stayed the same?
- In addition to the outcomes that directly relate to CYP, what changes have you seen in your workplace as a result of THRIVE? **Prompt:** for example has it made people’s jobs easier or harder? Have processes been streamlined? Has duplication been avoided? **Ask to give examples if says yes or no.**

**Organisation:**

*THRIVE leads:*

- Could you tell me how the specific goals of THRIVE are communicated to teams on the ground? I can give you some examples if you’ve gone blank!
- **Prompt:** Needs-based approach, not diagnosis/severity, shared accountability, communication between services, no wrong door…
- How satisfied do you feel with the structure of any implementation plans or timelines that you have been given? For example, do you feel that they are too rigid, too unstructured, or about right?
- **Prompt:** Is there anything you’d change about the plans? Why?

*Others:*

- How confident do you feel in your knowledge of the goals of THRIVE? How are these being communicated to you? **Further question:** Has this been sufficient?
- What new policies and/or procedures have been implemented in your workplace as a result of THRIVE?
- How well does everything you have learned about THRIVE fit in with your everyday duties? **Prompt:** do you have enough time/training/equipment?

**Other questions (elements of these have been captured with the NHS Sustainability Model, the below are additional questions that have not been covered):**

**Adaptability:**

*Top-down implementors:*

- I know you are conducting a number of surveys, with CYP about the service, and with staff about the training. Will the findings of these be actively used to make changes to develop and improve THRIVE? Will they be used to mould guidelines for the ongoing implementation?
- **Prompt:** How?
- Do you think, as a whole, THRIVE is a system that is adaptable and flexible, in the sense that it is open and responsive to new information, be it changing CYP needs, or feedback given by staff?
- **Prompt:** Can you give an example of how issues that arise are used to shape the system going forwards?
- Are guidelines/requirements adapted if significant barriers come up that are preventing staff from carrying them out?
- **Prompt:** How?
- What are the key elements of THRIVE that make it suitable for use with such a diverse population of CYP, and such a diverse range of services?

**Reflection:**

*Top-down implementors:*

- What do you think makes THRIVE different from other implementations that the NHS has been involved in?
- Have you had insight to any past implementations, that you have been able to use to guide the implementation of GM i-THRIVE?
- What were the key differences, or indeed similarities, between CAMHS processes and THRIVE principles that stood out to you when you began your role? What has been done to align these processes to THRIVE thus far? How successful do you think this has been?
- Do you have any examples of how the changes that have been made so far have been embedded into long-term policy?
- Have there been any negative or unintended consequences of implementing THRIVE, that you’ve seen in either formally through things like survey results, or from word of mouth?
- To what extent do you believe that the effort being put into implementing THRIVE matches, or will match, any benefits that will be seen in the long run?

*THRIVE leads:*

- What do you think makes THRIVE different from any other implementations that the NHS has been involved in?
- What were the key differences, or indeed similarities, between your locality’s previous processes and THRIVE principles that stood out to you when you began your role? What has been done in your locality to align these processes to THRIVE thus far? How successful do you think this has been?
- Do you know of any examples of how the changes that have been made so far have been embedded into long-term policy?
- Have there been any negative or unintended consequences of implementing THRIVE, that you’ve seen in your locality? Either formally, or through word of mouth?
- To what extent do you believe that the effort you are putting into implementing THRIVE matches, or will match, any benefits that your locality will see in the long run?

*Others:*

- In what ways does THRIVE feel different from other implementations or new ideas that you’ve experienced at work?
- Thinking back to how things were before THRIVE came along, what are the main things that have changed for you? **Prompt:** did you have any frustrations or problems that THRIVE has resolved? Are there any frustrations that still exist that you wish THRIVE had have resolved?
- Have you witnessed any negative consequences of THRIVE? **Prompt:** For example, reports from other staff or CYP/families, or perhaps any statistical changes that you know of?
- To what extent do you believe that the effort you are putting into implementing THRIVE matches, or will match, the benefits that your institution will see in the long run?

**Training:**

*Top-down implementors:*

- From your perspective, how does the i-THRIVE training academy actively promote the aims of GM i-THRIVE?
- How well do you think that the message is getting across? Could you give some examples of where you have seen sustainable practices taking place?
- How are gaps in staff’s knowledge and skills being identified? Is this process ongoing?
- Do you feel that there is enough staff capacity and support to carry out the new requirements? And have the new requirements altered job descriptions in any way?

*THRIVE leads:*

- From your perspective, how does the i-THRIVE training academy actively promote the aims of GM i-THRIVE?
- How well do you think that the message is getting across? Could you give some examples of where you have seen sustainable practices taking place?
- How are gaps in staff’s knowledge and skills being identified? Is this process ongoing?
- Do you feel that there is enough staff capacity and support to carry out the new requirements? And have the new requirements altered work schedules and job descriptions in any way?

*Others:*

- Did you take part in any of the GM i-THRIVE training academy sessions? Which one(s)?
- Did you find them useful? In what ways?
- **If did attend:** have you been able to share your knowledge from the training with your colleagues? Has much time been dedicated to this?
- **If didn’t:** what knowledge or information have you received from others who have attended some sessions? Was much time given to feeding this information down to you?
- Do you feel that you have enough capacity and support to carry out the new requirements? To your knowledge, has your work schedule and/or job description been altered at all as a result of the new requirements?

**Questionnaire:**

- Finally, I just wanted to know how you found the questionnaire I sent you, in terms of relevancy to you, applicability to your work, ease of understanding, etc.
